# Surgical Anatomy of the Distal Part of the Dorsal Scapular Nerve With a Focus on the Triple‐Tendon Transfer

**DOI:** 10.1002/brb3.70694

**Published:** 2025-07-11

**Authors:** Beyza Celikgun, Ozcan Gayretli, Ilke Ali Gurses, Osman Coskun, Aysin Kale

**Affiliations:** ^1^ Department of Anatomy, Faculty of Medicine Istanbul Health and Technology University Istanbul Turkey; ^2^ Department of Anatomy, Istanbul Faculty of Medicine Istanbul University Istanbul Turkey; ^3^ Department of Anatomy, Faculty of Medicine, Institute of Graduate Studies in Health Sciences Istanbul University Istanbul Turkey; ^4^ Department of Anatomy, Faculty of Medicine Koç University Istanbul Turkey

**Keywords:** dorsal scapular nerve, Eden–Lange, levator scapulae muscle, rhomboid muscles, triple tendon transfer

## Abstract

**Introduction:**

A review of the literature shows that most studies of the dorsal scapular nerve (DSN) have focused on morphological evaluation of the proximal part of the nerve. Morphometric studies contributing to clinical applications are limited. Therefore, we aimed to investigate the topographic and morphometric anatomy of the distal part of the DSN.

**Methods:**

13 cadavers in the Department of Anatomy, Istanbul Faculty of Medicine, were examined bilaterally. DSN dissection was performed on the anterior surface of the levator scapulae and rhomboids, and the distance to the medial border of the scapula (MBS) was recorded at different levels. The insertion lengths of the levator scapulae and rhomboids were also measured.

**Results:**

Two types of DSN were observed according to the level of termination in the muscle. Contrary to its classical course, the nerve ran laterally to the MBS on the four sides. The shortest distance between the nerve and the MBS was at the level of the superior border of the rhomboid minor (4.46 ± 9.88 mm). The insertion lengths of the levator scapulae and rhomboids according to gender and the insertion length of the rhomboid minor according to the level of termination in the muscle were significant.

**Discussion:**

We have obtained results that may be useful during Eden–Lange tendon transfer. The DSN is not always located medial to the MBS, it may be located lateral to it. To avoid nerve damage, we believe it is important to identify the nerve on the anterior surface of the muscles for a successful surgery.

## Graphical abstract

Two types of DSN were observed according to the level of termination in the muscle. Contrary to its classical course, DSN ran laterally to the MBS on the four sides. The shortest distance between DSN and MBS was at the level of the superior border of the rhomboid minor. The insertion lengths of the levator scapulae and rhomboids according to gender and the insertion length of the rhomboid minor according to the level of termination in the muscle were significant.

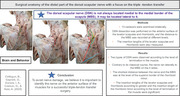

**Abbreviations**
DSN: Dorsal scapular nerve
MBS: Medial border of the scapula

## 1 Introduction

Due to its superficial location in the posterior cervical triangle, accessory spinal nerve injuries resulting from blunt trauma and traction injuries, cervical lymph node biopsies, or tumor excisions have been widely reported (Dunn 1974; Wright 1975; Battista 1991; Donner and Kline 1993; Bigliani et al. 1996; Teboul et al. 2004; Cesmebasi and Spinner 2015; Restrepo et al. 2015). Trapezius paralysis and atrophy as a result of accessory spinal nerve damage leads to shoulder drop and dysfunction, pain, and lateral scapular winging associated with impaired scapular movement.

Unsatisfactory clinical results of conservative treatments (Bigliani et al. 1985; Wiater and Bigliani 1999) and methods such as neurolysis, nerve repair, or nerve grafting have been reported to be less effective if not performed within the first 20 months after injury (Teboul et al. 2004; Göransson et al. 2016). Although most non‐penetrating injuries improve after 6–12 months of conservative treatment, it has been reported that some patients don't heal and in such cases, tendon transfers are the most appropriate treatment option (Dunn 1974; Wright 1975; Bigliani et al. 1985, 1999).

The Eden–Lange procedure was considered appropriate for the surgical treatment of trapezius paralysis resulting in lateral scapular winging, especially if it lasted longer than 20 months (Frontera et al. 2010). Due to the limited success of conservative management and the difficulty of nerve repair, tendon transfer, which is accepted as an effective treatment method for function restoration (Elhassan and Wagner 2015), was described by Eden in 1924 and was performed by transferring levator scapulae to the spine of the scapula and rhomboids to infraspinous fossa (Eden 1924). This technique, which was confirmed by Lange in the 1950s (Lange 1951) and named the Eden–Lange tendon transfer, was later modified by Bigliani et al. (1996) by transferring the rhomboid minor to the supraspinous fossa. In the following years, Elhassan and Wagner (2015), performed this technique as a triple tendon transfer by transferring all three muscles to the spine of the scapula.

The nerve that innervates these three transferred muscles is the dorsal scapular nerve (DSN). It is vulnerable to injury during the procedure. Therefore, it is very important to identify and protect the nerve (Martin and Fish 2008; Meininger et al. 2011). While there are several studies on the proximal part of the nerve (Tubbs et al. 2005; Nguyen et al. 2016; Tetsu et al. 2018; Jack et al. 2020; Williams and Smith 2020; Çelikgün et al. 2023), the number of morphometric studies examining how the distal part of the nerve is at risk in tendon transfers is limited. For this reason, we aimed to investigate the topographic and morphometric anatomy of the distal part of the DSN.

## 2 Methods

The DSN was dissected and examined in 26 specimens from 13 adult cadavers (11 male/2 female) in the Istanbul Faculty of Medicine, Department of Anatomy. The body donors ranged in age from 48 to 73 years old, with a mean age of 62 years. The cadavers had no history of trauma or surgical procedures in the neck and scapular regions. The authors state that every effort was made to follow all local and international ethical guidelines and laws that pertain to the use of human cadaveric donors in anatomical research (Iwanaga et al. 2022).

Each cadaver was placed in the prone position. The incision made up to the mastoid process was continued until the external occipital protuberance bilaterally. The next incision was made on the midline from the external occipital protuberance down to the sacrum. The third incision was made bilaterally from the spinous process of the vertebra prominens to the acromion. The fourth incision was made from the acromion to the middle of the axillary fossa, and finally, an incision was made bilaterally from the sacrum to the lateral part of the iliac crest.

Starting from the incision corners, the skin and superficial fascia were lifted laterally. The subcutaneous tissue was completely removed and the trapezius and latissimus dorsi muscle were revealed. The trapezius was released from its insertion and deviated medially, and the levator scapulae, rhomboid minor, and rhomboid major were exposed. The fascia on the muscles was cleaned, and the borders of the muscles and their insertions on the medial border of the scapula (MBS) were clarified.

**FIGURE 1:**
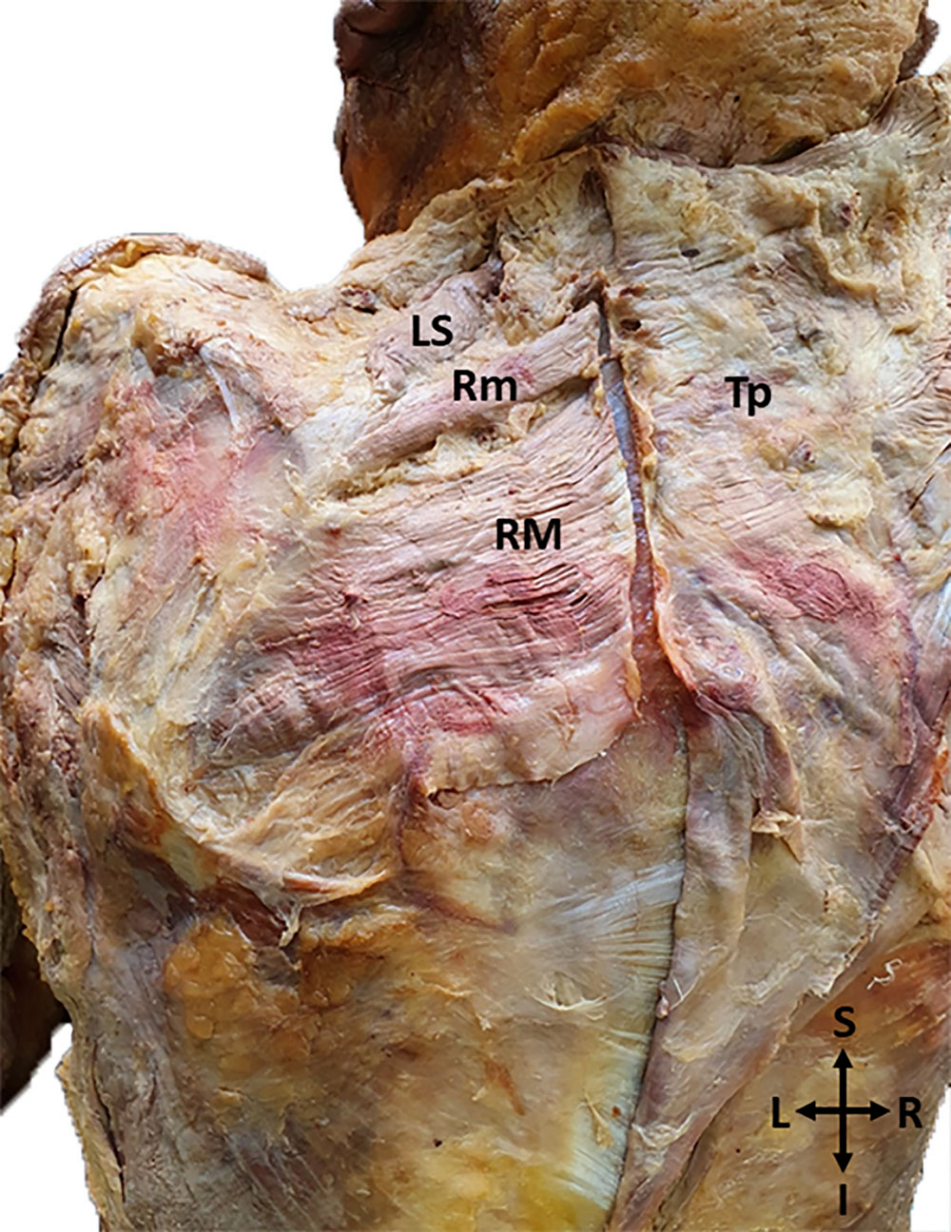
A photo of the levator scapulae and rhomboid muscles with exposure and incision rhomboids at their origins (I: Inferior; L: Left; LS: Levator scapulae; R: Right; RM: Rhomboid major; Rm: Rhomboid minor; S: Superior; Tp: Trapezius).

To follow the distal course of the DSN, which runs in the posteroinferiorly from its origin, on the anterior surface of the muscles; the rhomboids were cut from the origin and deviated laterally (Figure 1).

**FIGURE 2:**
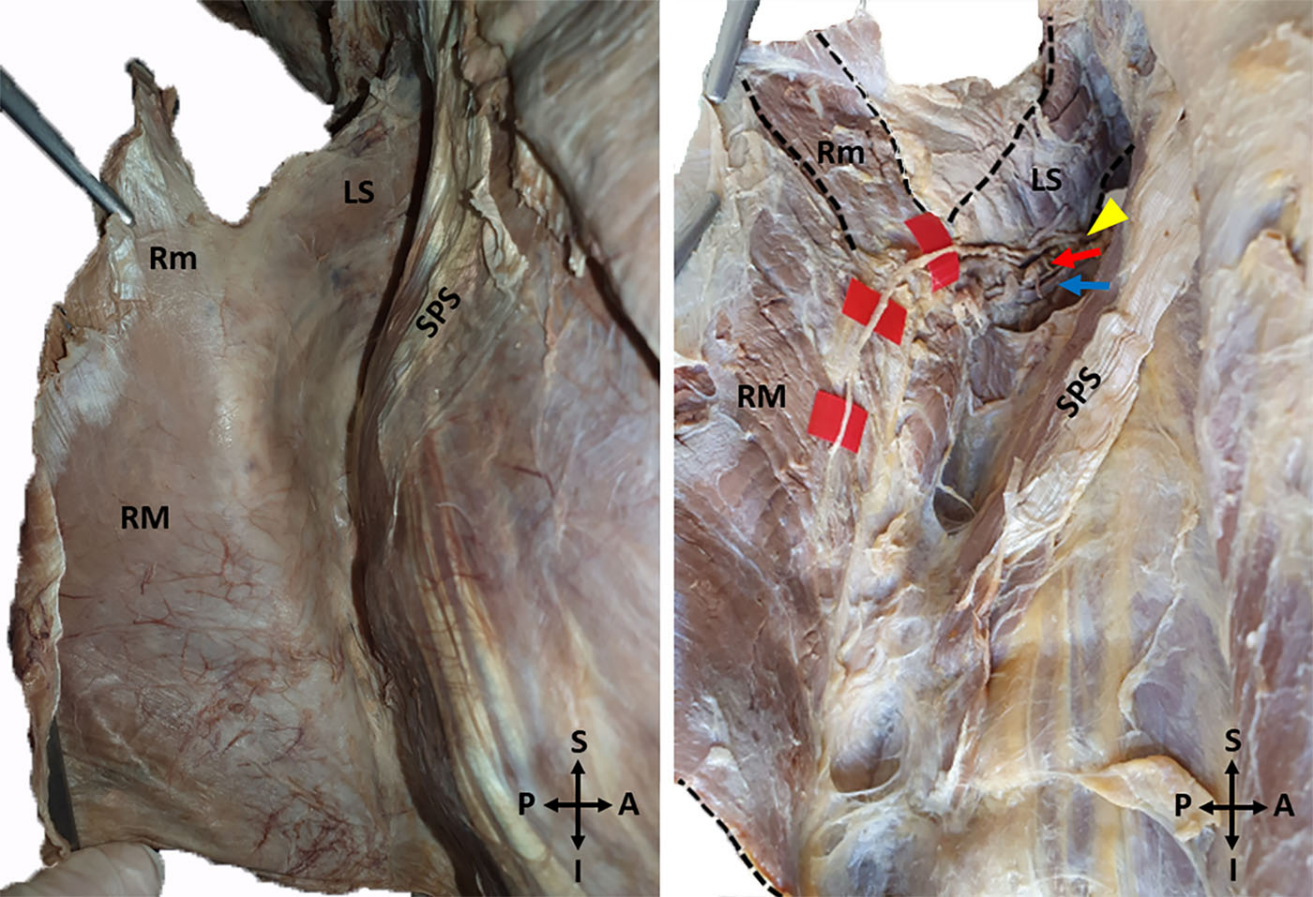
A photo of the tracing of the dorsal scapular nerve on the anterior surface of the muscles (Left side) (LS: Levator scapulae, Rm: Rhomboid minor, RM: Rhomboid major, SPS: Serratus posterior superior, Yellow arrowhead: Dorsal scapular nerve, Red arrow: Dorsal scapular artery, Blue arrow: Dorsal scapular vein, A: Anterior, P: Posterior, S: Superior, I: Inferior, Black dashed lines shows the boundaries of the muscles).

The fascia on the anterior surface of the rhomboids was carefully dissected. The DSN, which travels with the dorsal scapular artery and vein, was dissected to its termination in the muscle (Figure 2).

**FIGURE 3:**
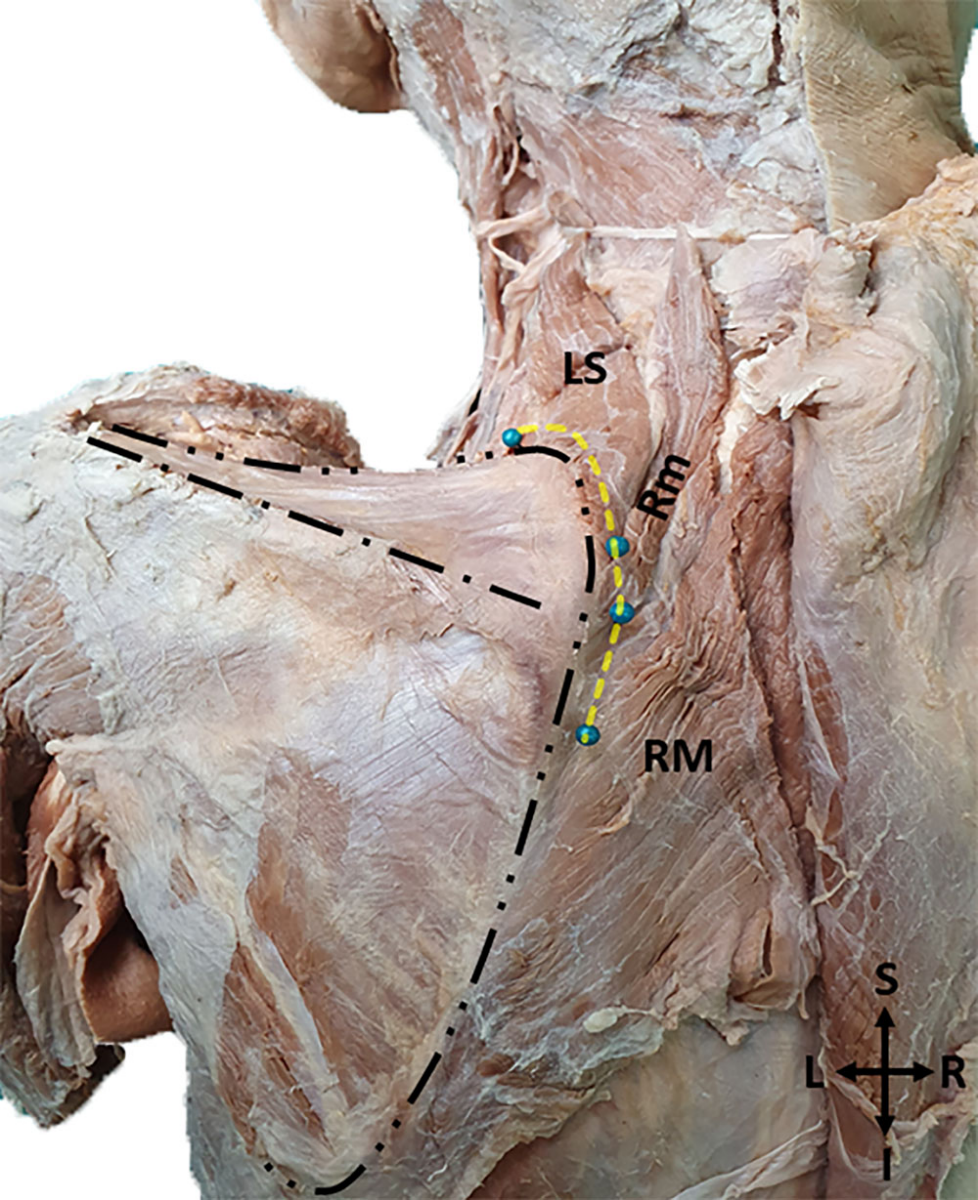
A photo of the marking of the dorsal scapular nerve course with pins on the posterior surface of the muscles (LS: Levator scapulae, Rm: Rhomboid minor, RM: Rhomboid major, R: Right, L: Left, S: Superior, I: Inferior. Yellow dashed line shows the course of the nerve, black dashed lines show the borders of the scapula and spine of the scapula.).

After the course of the nerve was revealed on the anterior surface of the muscles, to determine its distance from the MBS, the route of the nerve was marked with pins on the posterior surface of the muscles, and measurements were taken on this posterior surface (Figure 3).

**FIGURE 4:**
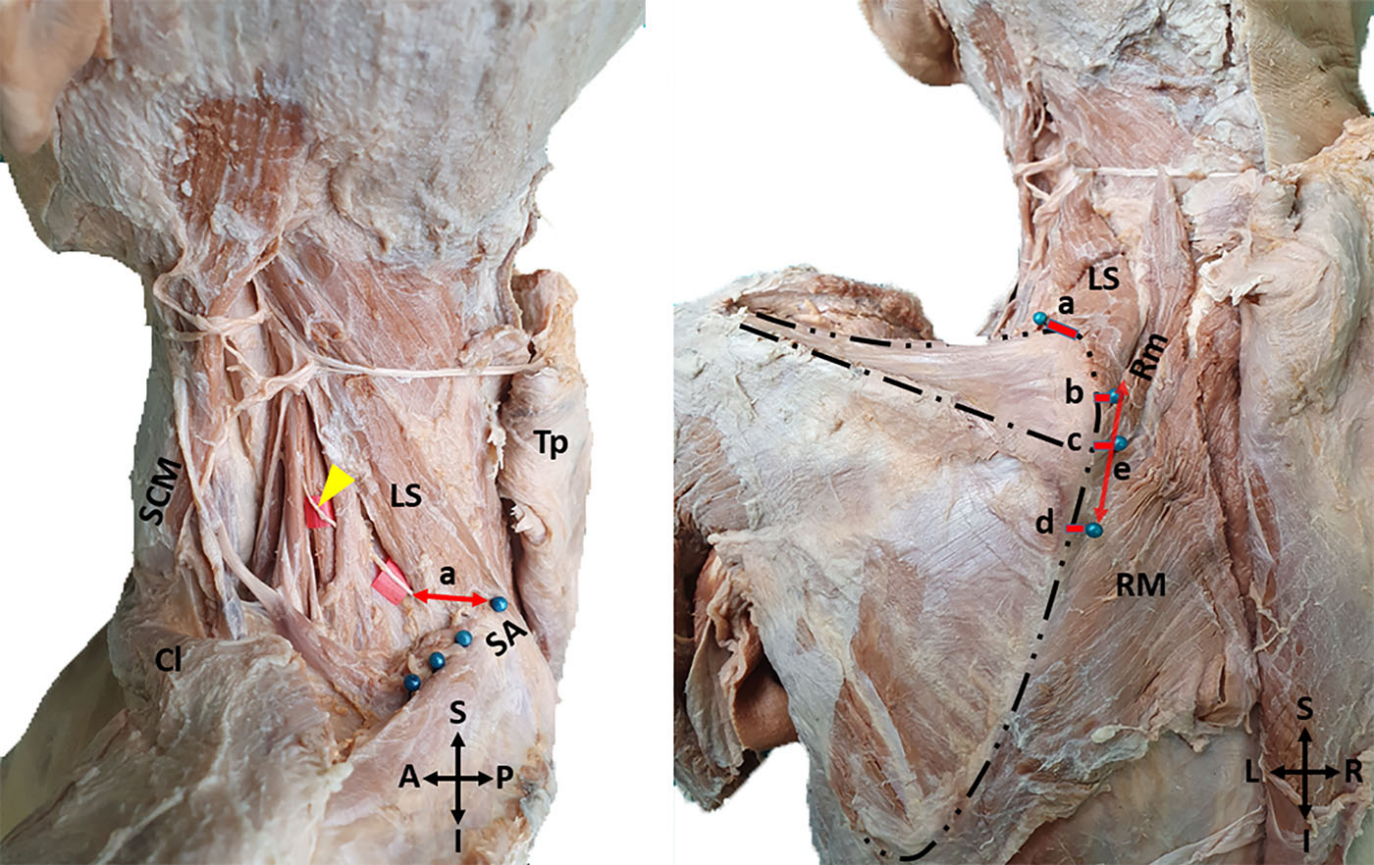
A photo of the measurements made for morphometric evaluation‐1 (Distance to the medial border of the scapula at the determined levels). **(Distance a**: The shortest distance between the point where the DSN reaches the anterior surface of the levator scapulae and the superior angle of the scapula. **Distance b**: The shortest distance between the point where the DSN reaches the anterior surface of the rhomboid minor and the MBS. **Distance c**: The shortest distance between the point where the DSN passes through the opening between the rhomboid minor and rhomboid major muscles and the MBS. The shortest distance of the DSN to the MBS at the level of the spine of the scapula. (In some of the cadavers, the point where the nerve passes through the opening between the rhomboid muscles and the point where it passes through the spina scapulae were at the same level, so both measurements are marked with the letter c). **Distance d**: The shortest distance between the level of DSN termination in the muscle and the MBS. **Distance e**: The shortest distance between the level of DSN termination in the muscle and the superior border of the rhomboid minor. (SCM: Sternocleidomastoid, Cl: Clavicula, LS: Levator scapulae, Tp: Trapezius, SA: Superior angle of the scapula, Yellow arrowhead: Dorsal scapular nerve, Rm: Rhomboid minor, RM: Rhomboid major, A: Anterior, P: Posterior, R: Right, L: Left, S: Superior, I: Inferior).

**FIGURE 5:**
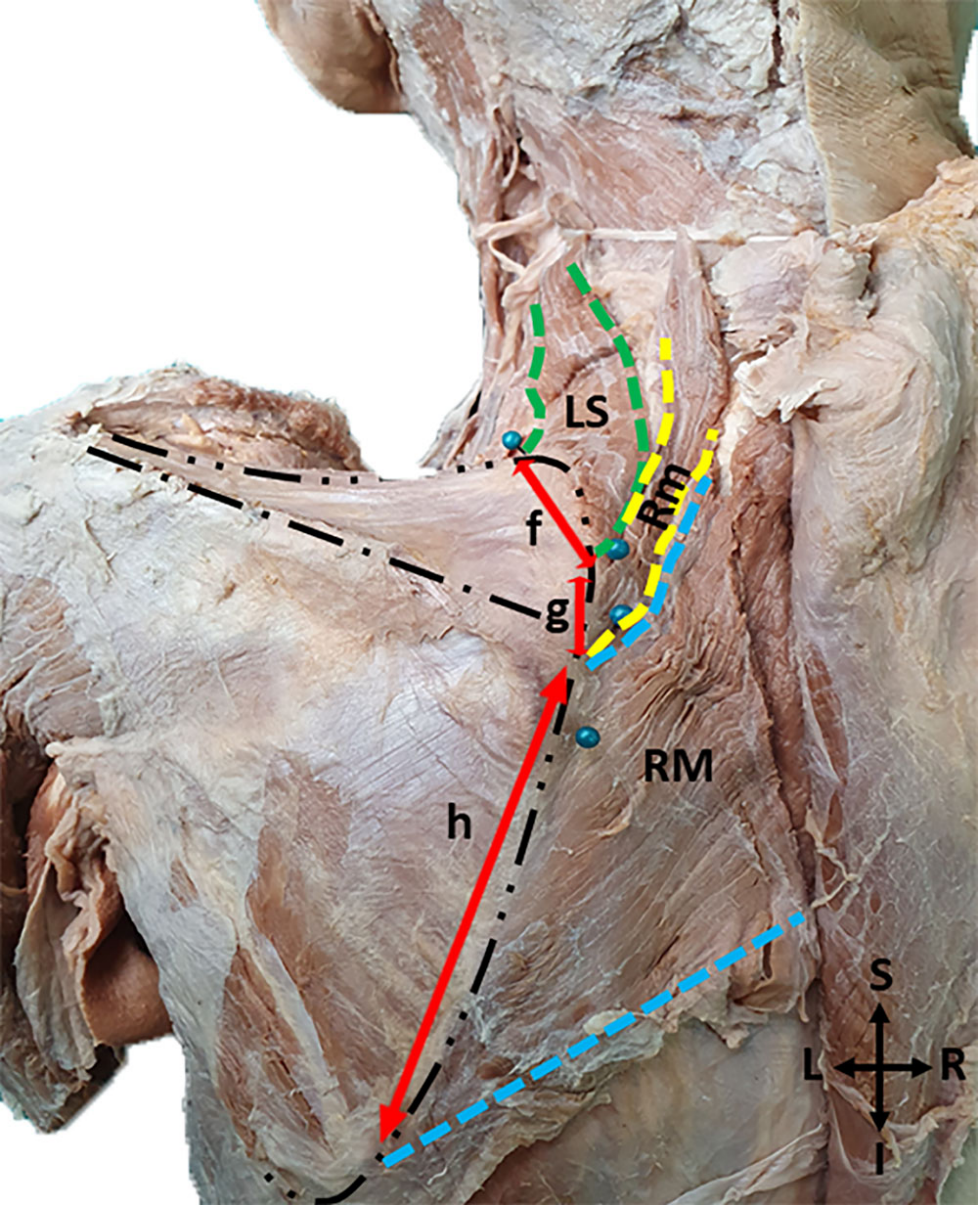
A photo of the measurements made for morphometric evaluation‐2 (The insertion lengths of the levator scapulae and rhomboids). (**Distance f**: The insertion length of the levator scapulae muscle. **Distance g**: The insertion length of the rhomboid minor muscle. **Distance h**: The insertion lengths of the rhomboid major muscle. LS: Levator scapulae, Rm: Rhomboid minor, RM: Rhomboid major R: Right, L: Left, S: Superior, I: Inferior. The coloured dashed lines show the boundaries of the muscles).

For morphological and morphometric evaluation:

- The shortest distance between the point where the DSN reaches the anterior surface of the levator scapulae and the superior angle of the scapula,
- The shortest distance between the point where the DSN reaches the anterior surface of the rhomboid minor and the MBS,
- The shortest distance between the point where the DSN passes through the opening between the rhomboid minor and rhomboid major muscles and the MBS,
- The shortest distance of the DSN to the MBS at the level of the spine of the scapula,
- The level of DSN termination in the muscle,
- The shortest distance between the level of DSN termination in the muscle and the MBS,
- The shortest distance between the level of DSN termination in the muscle and the superior border of the rhomboid minor,
- The insertion lengths of the levator scapulae and rhomboids were examined (Figures 4 and 5).

All measurements were made bilaterally with a digital caliper (Mitutoyo Company, Kawasaki‐shi, Kanagawa, Japan). The shortest distance was taken as a reference. Measurements were repeated three times by the same researcher, and the average of the measurements was recorded.

Statistical Package for the Social Sciences software v23.0 for Windows (SPSS, Inc., Chicago, IL, USA) was used for statistical analysis and interpretation of the obtained data. Whether the data showed normal distribution or not was evaluated with Kolmogorov–Smirnov and Shapiro–Wilk tests. Numerical data were expressed as mean and standard deviation and categorical data as percentages. T‐test was used for comparison between binary groups for parameters with normal distribution. ANOVA test was used to compare three or more groups with normal distribution. The chi‐square test, one of the parametric tests, was applied to continuous variables with normal distribution. The results were evaluated at a 95% confidence interval and *p* < 0.05 significance level.

## 3 Results

### 3.1 The Level of DSN Termination in the Muscle

**FIGURE 6:**
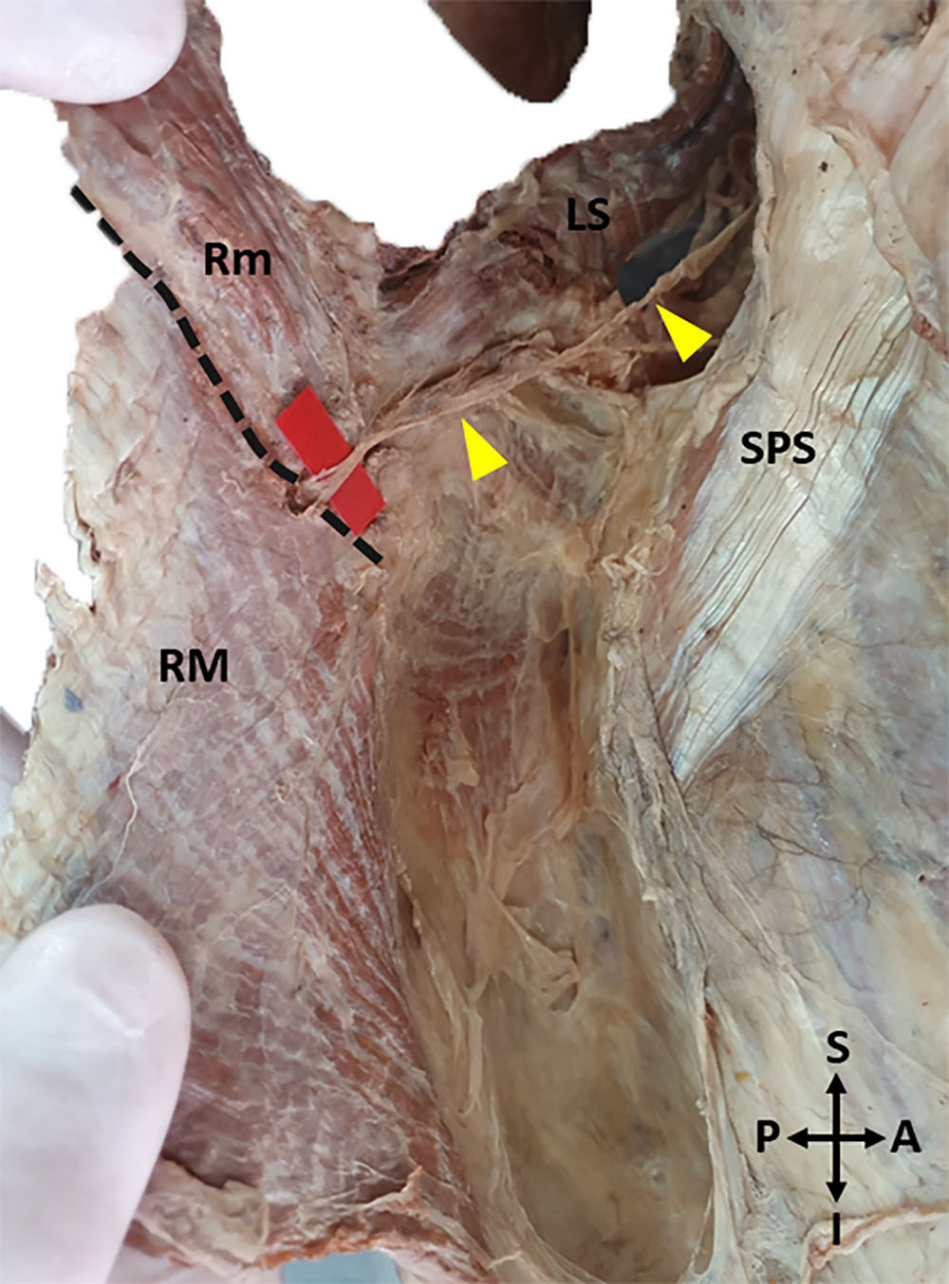
A photo of the type of the dorsal scapular nerve entering the muscle from the superior border of the rhomboid major (Left side). (LS: Levator scapulae, Yellow arrowheads: Dorsal scapular nerve, Rm: Rhomboid minor, RM: Rhomboid major, SPS: Serratus posterior superior, A: Anterior, P: Posterior, S: Superior, I: Inferior).

**FIGURE 7:**
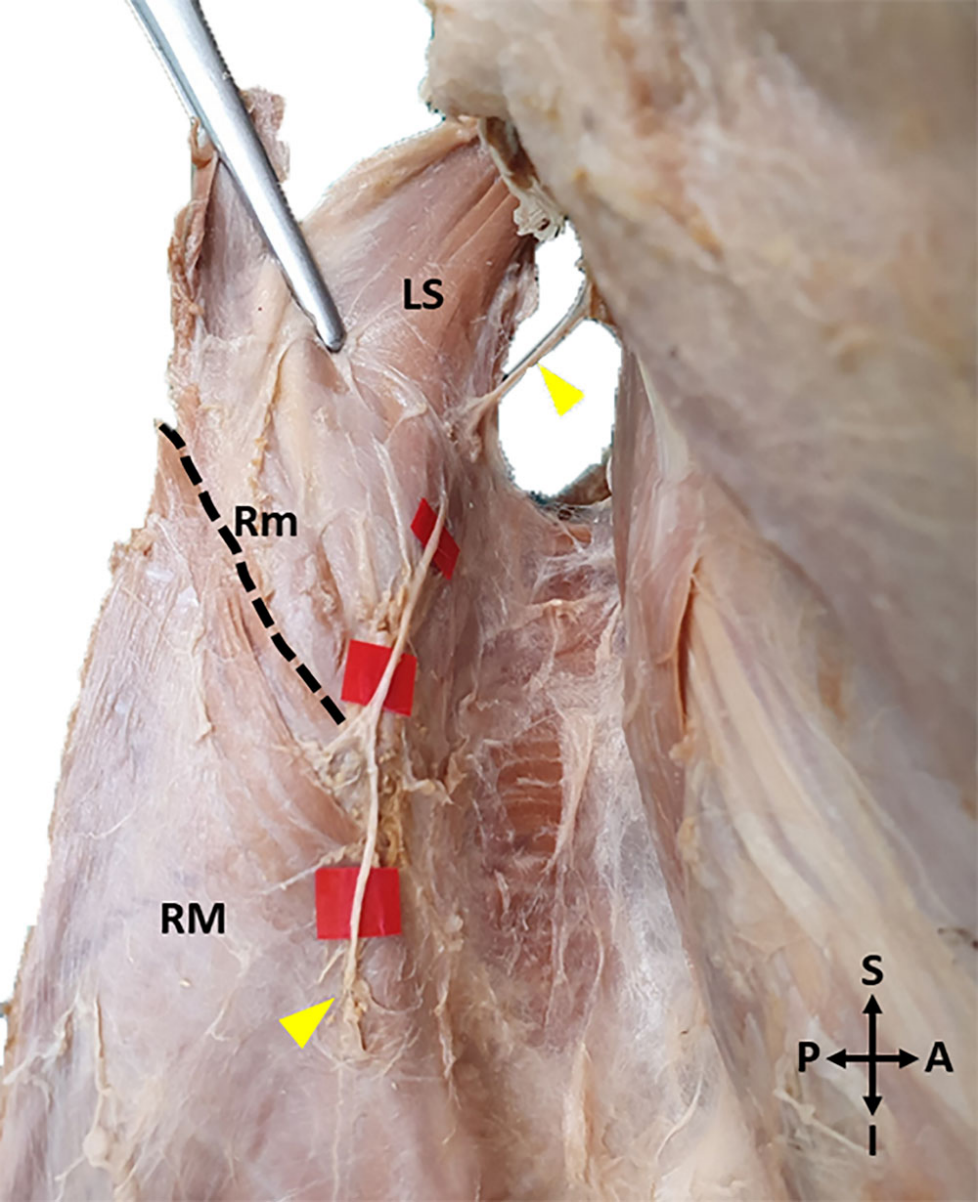
A photo of the type of the dorsal scapular nerve entering the muscle from the anterior surface of the rhomboid major (Left side) (LS: Levator scapulae, Yellow arrowheads: Dorsal scapular nerve, Rm: Rhomboid minor, RM: Rhomboid major, A: Anterior, P: Posterior, S: Superior, I: Inferior. Black dashed line shows the superior border of the rhomboid major).

Two types of DSN were seen according to the level of DSN termination in the muscle. Around 65.4% (17 sides) of the DSNs entered the muscle from the superior border of the rhomboid major. Around 34.6% (9 sides) of the DSNs traveled more distally and entered the muscle from the anterior surface of the rhomboid major (Figures 6 and 7).

### 3.2 Position of the DSN Relative to the Medial Border of the Scapula

**FIGURE 8:**
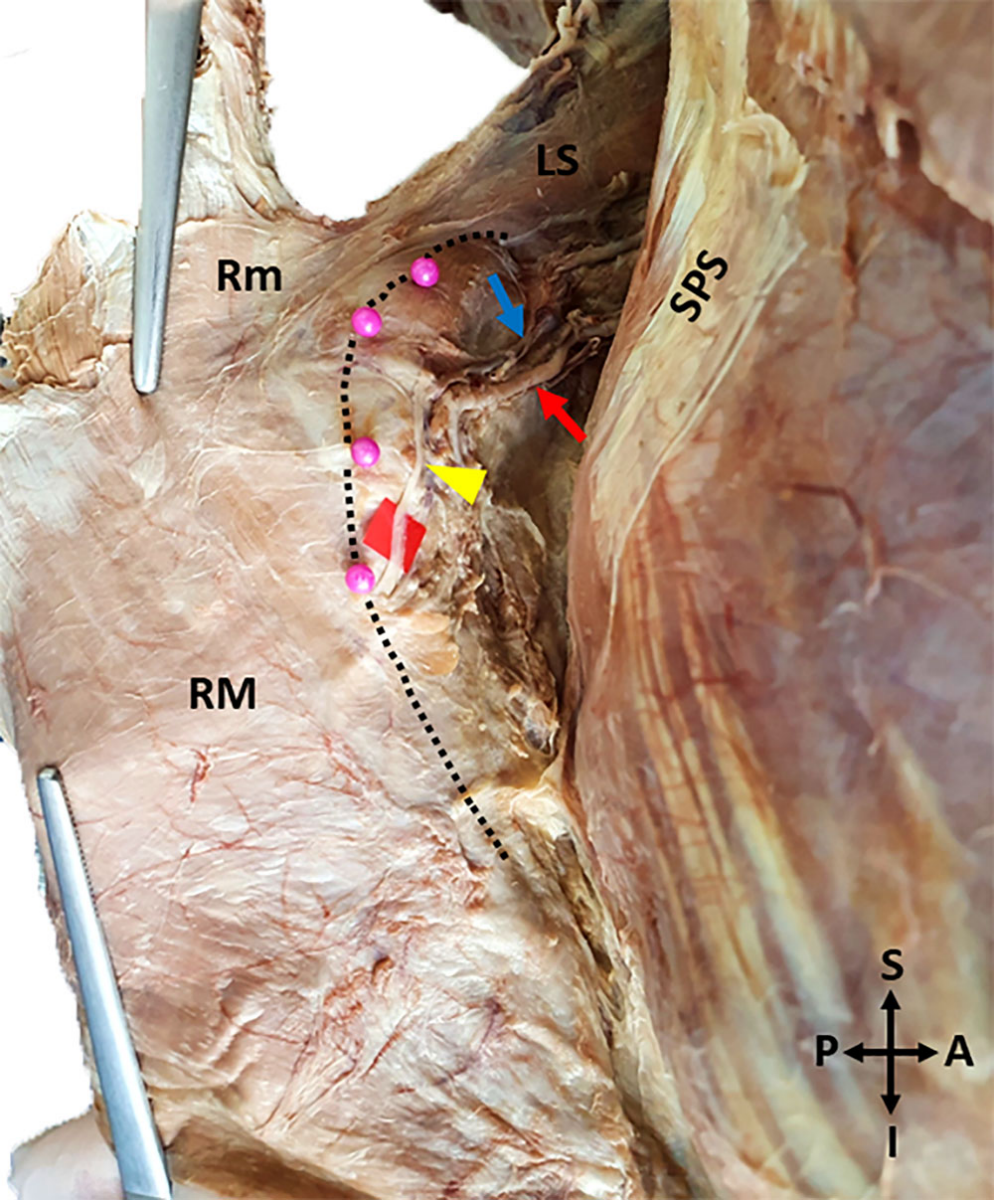
A photo of the dorsal scapular nerve travelling lateral to the medial border of the scapula (Left side) (LS: Levator scapulae, Rm: Rhomboid minor, RM: Rhomboid major, SPS: Serratus posterior superior, Pink pinheads and black dashed line: Medial border of the scapula, Yellow arrowhead: Dorsal scapular nerve, Red arrow: Dorsal scapular artery, Blue arrow: Dorsal scapular vein, A: Anterior, P: Posterior, S: Superior, I: Inferior).

The DSN traveled lateral to the MBS, contrary to its classical anatomical course in 2 male cadavers unilaterally, and in 1 male cadaver bilaterally (Figure 8).

### 3.3 Morphometric Evaluation

Statistics of DSN (a–e) and musculoskeletal measurements (f–h) are summarized in Tables 1 and 2.

**TABLE 1:** A table that shows statistics of dorsal scapular nerve measurements.

Measurement	Mean ± SD (mm)					Range (mm) (Total)
	Total	Female	Male	Right side	Left side	
**Distance a**	(*N* = 26) 13.09 ± 9.77	(*N* = 4) 12.37 ± 9.91	(*N* = 22) 13.22 ± 9.97	(*N* = 13) 12.93 ± 11.95	(*N* = 13) 13.25 ± 7.47	−2.9^*^–38.5
**Distance b**	(*N* = 26) 4.46 ± 9.88	(*N* = 4) 5.95 ± 5.49	(*N* = 22) 4.19 ± 10.55	(*N* = 13) 6.03 ± 10.67	(*N* = 13) 2.88 ± 9.17	−19.6^*^–18.9
**Distance c¹**	(*N* = 11) 9.48 ± 6.27	(*N* = 2) 11.1 ± 4.1	(*N* = 9) 9.12 ± 6.8	(*N* = 7) 10.08 ± 4.29	(*N* = 4) 8.42 ± 9.58	−4.3^*^–17.4
**Distance c^2^**	(*N* = 18) 9.08 ± 10.29	(*N* = 4) 12.82 ± 7.48	(*N* = 14) 8.0 ± 10.96	(*N* = 8) 7.61 ± 12.22	(*N* = 10) 10.25 ± 8.97	−20.1^*^–22.8
**Distance d**	(*N* = 26) 9.35 ± 10.88	(*N* = 4) 13.9 ± 6.88	(*N* = 22) 8.53 ± 11.38	(*N* = 13) 7.87 ± 11.64	(*N* = 13) 10.84 ± 10.3	−18.0^*^–22.8
**Distance e**	(*N* = 26) 32.5 ± 20.18	(*N* = 4) 23.15 ± 6.47	(*N* = 22) 34.19 ± 21.43	(*N* = 13) 32.53 ± 21.77	(*N* = 13) 32.46 ± 19.35	2.9–83.0

***Note***: Distance a: The shortest distance between the point where the DSN reaches the anterior surface of the levator scapulae and the superior angle of the scapula; Distance b: The shortest distance between the point where the DSN reaches the anterior surface of the rhomboid minor and the MBS; Distance c¹: The shortest distance between the point where the DSN passes through the opening between the rhomboid minor and rhomboid major muscles and the MBS; Distance c^2^: The shortest distance of the DSN to the MBS at the level of the spine of the scapula (In some of the cadavers, the point where the nerve passes through the opening between the rhomboid muscles and the point where it passes through the spina scapulae were at the same level, so both measurements are marked with the letter c); Distance d: The shortest distance between the level of DSN termination in the muscle and the MBS; Distance e: The shortest distance between the level of DSN termination in the muscle and the superior border of the rhomboid minor.

Abbreviations: N, sample size; SD, standard deviation.

* The measurement value was recorded as negative data in the samples located lateral to the scapula.

**TABLE 2:** A table that shows statistics of musculoskeletal measurements.

Measurement	Mean ± SD (mm)					Range (mm) (Total)
	Total	Female	Male	Right side	Left side	
**Distance f**	(*N* = 26) 36.63 ± 3.96	(*N* = 4) 31.47 ± 4.23	(*N* = 22) 37.57 ± 3.19	(*N* = 13) 37.15 ± 3.04	(*N* = 13) 36.11 ± 4.79	25.8–43.2
**Distance g**	(*N* = 26) 23.1 ± 5.73	(*N* = 4) 18.55 ± 2.66	(*N* = 22) 23.93 ± 5.78	(*N* = 13) 22.93 ± 6.46	(*N* = 13) 23.27 ± 5.16	13.3–41.8
**Distance h**	(*N* = 26) 95.68 ± 14.97	(*N* = 4) 80.35 ± 3.79	(*N* = 22) 98.47 ± 14.55	(*N* = 13) 95.28 ± 13.18	(*N* = 13) 96.08 ± 17.11	62.0–118.6

***Note***: Distance f: The insertion length of the levator scapulae muscle; Distance g: The insertion length of the rhomboid minor muscle; Distance h: The insertion length of the rhomboid major muscle.

Abbreviations: N, sample size; SD, standard deviation.

There was no significant difference in side comparisons with statistical tests but the insertion lengths of the levator scapulae (*p* = 0.008), rhomboid minor (*p* = 0.028), and rhomboid major (*p* = 0.033) muscles were found to be significantly longer in males than females.

In addition, in cases where the DSN entered the muscle from the superior border of the rhomboid major muscle, the insertion length of the rhomboid minor muscle was found to be longer with a statistically significant difference (25.07 ± 5.87 mm, *p* = 0.013)

## 4 Discussion

### 4.1 Variations of Muscle Innervation of the DSN

Although the muscles typically innervated by the DSN are known as the levator scapulae, rhomboid minor, and rhomboid major (Schuenke et al. 2014; Tubbs et al. 2015; Standring 2016; Moore et al. 2018), several variations have been reported.

In the case report study of Kida and Tani (1993), it was reported that the serratus posterior superior muscle was innervated by both the intercostal nerves and the DSN.

In the study of Frank et al. (1997), 35 sides were examined and it was found that the DSN innervates the rhomboid muscles on all sides, but not the levator scapulae muscle on 24 sides. This study also found that the DSN pierced the levator scapulae muscle on 9 sides.

The studies by Tubbs et al. (2005) and Tetsu et al. (2018) examined 10 and 70 cadavers respectively and found that the levator scapulae and the rhomboid muscles were innervated bilaterally by the DSN on all sides.

In the study of Nguyen et al. (2016), 23 sides were examined and it was found that the DSN innervates the levator scapulae muscle on all sides, but not the rhomboid muscles on 11 sides.

In our study, we observed that the DSN innervates the levator scapulae and the rhomboid muscles in all 26 sides we examined. In addition, we observed that the DSN pierced the levator scapulae muscle unilaterally in 2 cadavers, instead of traveling ventrally to the muscle. We think that such a variation should be taken into account, as it may trap the nerve in the levator scapulae and cause symptoms.

### 4.2 The Level of DSN Termination in the Muscle

There is no study in the literature on this topic. In our study, 65.4% (17 sides) of the DSNs entered the muscle from the superior border of the rhomboid major, and 34.6% (9 sides) of the DSNs entered the muscle from the anterior surface of the rhomboid major.

Clinically, trapezius muscle paralysis and atrophy as a result of spinal accessory nerve injury. In the Eden–Lange triple tendon transfer used to treat this condition, the position of the DSN relative to the MBS and its distal course is very important.

In the Eden–Lange procedure, which is used in the surgical treatment of trapezius paralysis, the levator scapulae and rhomboid muscles are transferred (Eden 1924; Lange 1951; Bigliani et al. 1996; Elhassan and Wagner 2015; Gustin et al. 2020). Although some variations have been reported, the DSN, which innervates mostly all three muscles, is vulnerable to injury during transfer procedures. There are no reported cases of DSN injury during tendon transfer in the literature, but the nerve is still at risk, so it is very important to determine the course of the nerve.

Therefore, we believe it is important to determine the course and the termination level of the nerve in order to prevent iatrogenic nerve damage and achieve successful surgical results.

### 4.3 Position of the DSN Relative to the Medial Border of the Scapula

The DSN typically travels posteroinferiorly after piercing the middle scalene muscle, between the levator scapulae and the posterior scalene muscle, on the anterior surface of the rhomboids and medial to the MBS (Moses et al. 2013; Schuenke et al. 2014; Tubbs et al. 2015; Standring 2016; Moore et al. 2018).

Tubbs et al. (2005) examined 10 cadavers bilaterally and reported that the DSN travels approximately 15 mm (range: 10–32 mm) medial to the MBS.

Pinto et al. (2019) examined 20 sides of 12 cadavers and, as in our study, performed measurements that shed light on the surgical anatomy of the DSN, which is important for Eden–Lange tendon transfer and recorded the distance of the nerve to the MBS at different points.

In the study by Pinto et al. (2019), the mean distance between the point where the DSN reaches the anterior surface of the levator scapulae and the superior angle of the scapula was found to be 27.6 ± 9.1 mm, in our study, this distance was 13.09 ± 9.77 mm.

In the study by Pinto et al. (2019), the mean distance between the point where the DSN reaches the anterior surface of the rhomboid minor and the MBS was found to be 16.1 ± 5.3 mm, in our study, this distance was 4.46 ± 9.88 mm.

In a study performed by Goubier and Teboul (2015) on 10 sides of 5 cadavers to evaluate the suitability of transferring the terminal part of the DSN to the suprascapular nerve, it was reported that the nerve should be cut just above the upper edge of the rhomboid minor to obtain maximum diameter and length. The results obtained in this parameter of our study may also be important during the transfer of the terminal part of the DSN to the suprascapular nerve, except for the Eden–Lange tendon transfer.

In the study by Pinto et al. (2019), the mean distance between the point where the DSN passes through the opening between the rhomboid minor and rhomboid major muscles and the MBS was found to be 18.1 ± 3.9 mm, in our study, this distance was 9.48 ± 6.27 mm.

There is no study in the literature on the distance of the DSN to the MBS at the level of the spine of the scapula. In our study, the mean value of this distance was measured as 9.08 ± 10.29 mm. As it is a palpable anatomical point, we believe that the distance of the nerve to the MBS at the level of the spine of the scapula may be an important reference in surgery and further studies are needed on the position of the nerve at this point.

In the study by Pinto et al. (2019), the mean distance between the level of DSN termination in the muscle and the MBS was found to be 22.9 ± 6.9 mm, in our study, this distance was 9.35 ± 10.88 mm.

In the study by Pinto et al. (2019), the mean distance between the level of DSN termination in the muscle and the superior border of the rhomboid major was found to be 37.5 ± 27 mm. In our study, it was observed that the DSN mostly terminated at the level of the superior border of the rhomboid major and therefore the distance from the level of the nerve termination to the superior border of the rhomboid minor was measured. The mean value of this distance was 32.5 ± 20.8 mm.

Pinto et al. (2019) reported that the DSN was located closest to the MBS at the superior border of the rhomboid minor on 11 of the 20 sides, at the opening between the rhomboid muscles on 5 sides, and at the termination level of the nerve in 3 sides. On 1 side, it was reported that the nerve traveled equidistant to the MBS at these three levels.

In our study, the nerve was found to be closest to the MBS at the superior border of the rhomboid minor in 12 of the 24 sides, at the level where the DSN reached the anterior surface of the levator scapulae in 7 sides, at the opening between the rhomboid muscles in 4 sides, and at the termination level of the nerve in 3 sides.

Pinto et al. (2019) found the minimum distance between the DSN and the MBS to be 7 mm in the cadavers they examined and reported that this distance should be taken into consideration when separating the muscles from their insertions so that the Eden–Lange tendon transfer can be completed without damaging the nerve. In our study, the DSN was observed to run along the MBS at the level where the DSN reached the anterior surface of the levator scapulae in 1 side and at the level where the DSN reached the anterior surface of the rhomboid minor on 1 side.

In addition, Pinto et al. (2019) reported that the DSN didn't cross the MBS in any of the cadavers and therefore the nerve wouldn't be damaged if the muscles were separated directly from their scapular insertion during triple tendon transfer. In our study, it was observed that the DSN traveled lateral to the MBS at the superior border of the rhomboid minor on 7 sides, at the level where the nerve was intramuscular on 3 sides, at the level of the spine of the scapula on 2 sides, and at the opening between the rhomboids on 1 side.

Therefore, the nerve may not always be located on the medial to the MBS; but may run directly over the edge or lateral to it. So far, no case of the DSN injury during this transfer has been reported in the literature. However, we believe that the nerve should be visualized and separated from the insertions of the muscles according to its course in order to avoid damage.

Furthermore, Pinto et al. (2019) stated that the level where the DSN was closest to the MBS was the upper edge of the rhomboid minor and therefore, this level was the most dangerous zone for tendon transfer. Similarly, in our study, this level was observed as the closest DSN distance to the MBS. We believe that more care is needed when performing tendon transfers, especially at this level.

### 4.4 Insertion Lengths of Muscles

In the study by Pinto et al. (2019), the mean insertion length of the levator scapulae was found to be 37.5 ± 8.5 mm, in our study, this measurement was 36.63 ± 3.96 mm. In the study by Goubier and Teboul (2015), which evaluated the suitability of transferring the terminal portion of the DSN innervating the rhomboids to the suprascapular nerve, it was noted that the levator scapulae was cut at the point of insertion into the scapula to expose the branch of the DSN to the rhomboids. The results we obtained may be important for the transfer of the DSN to the suprascapular nerve in addition to Eden–Lange tendon transfer.

In the study by Pinto et al. (2019), the mean insertion length of the rhomboid minor was found to be 18.7 ± 5.7 and 106.6 ± 17.6 mm for rhomboid major. In our study, these measurements were 23.1 ± 5.73 mm for rhomboid minor and 95.68 ± 14.97 mm for rhomboid major.

## 5 Conclusion

In our study, we obtained results that may be useful during Eden–Lange triple tendon transfer for restoration of trapezius function. The DSN is not always located medial to the MBS, it may be located lateral to it or directly over it. In order to avoid nerve damage, we believe it is important to identify the DSN on the anterior surface of the muscles for a successful surgery.

We believe that the results of our study contribute to the literature and play a role in reducing the complications that may occur during clinical applications and increasing the success of treatment.

## Author Contributions

**Beyza Celikgun**: conceptualization, investigation, data curation, methodology, visualization, writing–original draft, writing–review and editing. **Ozcan Gayretli**: investigation, project administration, resources, supervision, validation, writing–review and editing. **Ilke Ali Gurses**: conceptualization, data curation, formal analysis, investigation, methodology. **Osman Coskun**: supervision, resources, visualization, writing–review and editing. **Aysin Kale**: resources, supervision, writing–review and editing, validation.

## Ethics Statement

We confirm that we have read the journal's position on issues involved in ethical publication and affirm that this report is consistent with those guidelines.

## Conflicts of Interest

The authors declare no conflicts of interest.

## Peer Review

The peer review history for this article is available at https://publons.com/publon/10.1002/brb3.70694

## Data Availability

The data that support the findings of this study are available on request from the corresponding author. The data are not publicly available due to privacy or ethical restrictions.
